# Robust stratification of breast cancer subtypes using differential patterns of transcript isoform expression

**DOI:** 10.1371/journal.pgen.1006589

**Published:** 2017-03-06

**Authors:** Thomas P. Stricker, Christopher D. Brown, Chaitanya Bandlamudi, Megan McNerney, Ralf Kittler, Vanessa Montoya, April Peterson, Robert Grossman, Kevin P. White

**Affiliations:** 1 Institute for Genomics and Systems Biology, University of Chicago, Chicago, IL, United States of America; 2 Department of Pathology, Microbiology and Immunology, Vanderbilt University Medical Center, Nashville, TN, United States of America; 3 Department of Genetics, University of Pennsylvania, Philadelphia, PA, United States of America; 4 Department of Pathology, University of Chicago, Chicago, IL, United States of America; 5 McDermott Center for Human Growth and Development, University of Texas Southwestern, Dallas, TX, United States of America; 6 Cancer Biology and Epigenomics Program, Ann and Robert H. Lurie Children’s Hospital of Chicago Research Center, Chicago, IL, United States of America; 7 Laboratory of Genetics, University of Wisconsin, Madison, WI, United States of America; 8 Department of Medicine, University of Chicago, Chicago, IL, United States of America; 9 Department of Human Genetics, University of Chicago, Chicago, IL, United States of America; 10 Tempus Labs, Inc. Chicago, IL 60654, United States of America; National Cancer Institute, UNITED STATES

## Abstract

Breast cancer, the second leading cause of cancer death of women worldwide, is a heterogenous disease with multiple different subtypes. These subtypes carry important implications for prognosis and therapy. Interestingly, it is known that these different subtypes not only have different biological behaviors, but also have distinct gene expression profiles. However, it has not been rigorously explored whether particular transcriptional isoforms are also differentially expressed among breast cancer subtypes, or whether transcript isoforms from the same sets of genes can be used to differentiate subtypes. To address these questions, we analyzed the patterns of transcript isoform expression using a small set of RNA-sequencing data for eleven Estrogen Receptor positive (ER+) subtype and fourteen triple negative (TN) subtype tumors. We identified specific sets of isoforms that distinguish these tumor subtypes with higher fidelity than standard mRNA expression profiles. We found that alternate promoter usage, alternative splicing, and alternate 3’UTR usage are differentially regulated in breast cancer subtypes. Profiling of isoform expression in a second, independent cohort of 68 tumors confirmed that expression of splice isoforms differentiates breast cancer subtypes. Furthermore, analysis of RNAseq data from 594 cases from the TCGA cohort confirmed the ability of isoform usage to distinguish breast cancer subtypes. Also using our expression data, we identified several RNA processing factors that were differentially expressed between tumor subtypes and/or regulated by estrogen receptor, including YBX1, YBX2, MAGOH, MAGOHB, and PCBP2. RNAi knock-down of these RNA processing factors in MCF7 cells altered isoform expression. These results indicate that global dysregulation of splicing in breast cancer occurs in a subtype-specific and reproducible manner and is driven by specific differentially expressed RNA processing factors.

## Author summary

Breast cancer, the second leading cause of cancer death of women worldwide, is a heterogenous disease. Different subtypes of breast cancer display very different expression programs, and these expression programs are associated with different patient outcomes and with different treatment protocols. However, little is known about what drives these subtype differences. By sequencing RNA in a discovery cohort of breast cancer patients, we demonstrate that different subtypes of breast cancer can be distinguished by simply using differential transcript isoform expression. We confirmed our findings using two additional patient cohorts. We also demonstrate that differential expression of RNA processing factors between subtypes can affect differences in isoform usage. Using RNAi we knock down differentially expressed RNA processing factors including YBX1, YBX2, MAGOH, MAGOHB, and PCBP2, and show that this knock-down results in differential isoform expression of the genes identified in our disease subtype panel. Taken together, our results indicate that global dysregulation of splicing occurs in a subtype-specific and reproducible manner in breast cancer, and is driven by specific differentially expressed RNA processing factors.

## Introduction

Breast cancer is the most common carcinoma in women world-wide and is the second leading cause of cancer death in American women [1]. The vast majority of these tumors are adenocarcinomas that develop from the mammary epithelium. Pathologists categorize breast tumors based on expression of the estrogen and progesterone receptors (ER, PR, respectively) and amplification of ERBB2 (Her2Neu) [2]. These pathologic categories determine therapy and suggest prognosis; tumors that are positive for ER frequently respond to drugs that antagonize ER, while tumors with ERBB2 amplification respond to transtuzumab [3–5]. Tumors that lack expression of ER, PR, and lack amplification of ERBB2 are known as triple negative tumors; these tumors represent a major clinical challenge, as they lack targeted therapies and are treated with standard chemotherapy [6,7]. Although many triple negative breast cancers are initially responsive to chemotherapy, they have a worse 4-year distant disease free and overall survival due to an increased relapse rate amongst those with residual disease and account for a disproportionate number of breast cancer related deaths [6,7]. This clinical heterogeneity is reflected in molecular expression profiles, as expression profiling has established five categories of ductal breast carcinomas that carry different prognoses and have different survivals: 1) Luminal A, 2) Luminal B, 3) Her2(+), 4) normal and 5) basal-like (triple negative) subtypes [8]. These molecular expression profiles largely, but not completely, overlap with the estrogen, progesterone, and ERBB2 status of tumors [8].

Studies establishing the molecular subtypes of breast cancer demonstrated the global dysregulation of the breast cancer transcriptome. However, microarrays do not accurately capture variation in isoform usage. Dysregulation of differential splicing of the transcriptome has emerged as an important phenomenon in tumorigenesis [9,10][11][12]. Indeed, several genes critical to breast cancer, such as TP53, BRCA1, PTEN, and CD44, have been shown to have cancer-specific splice isoforms [13–15]. Furthermore, alternative splicing of CD44 and several other genes in breast cancer cell lines contributes to the epithelial-to-mesenchymal switch and may promote metastasis [14,15][16].

RNA sequencing allows for efficient and accurate assessment of isoform usage [17,18]. To determine whether alternative splicing differs between breast cancer subtypes, we used whole transcriptome sequencing to identify differentially expressed genes and differentially expressed transcript isoforms, from eleven ER+ and fourteenTN breast cancers. We confirm that gene expression variation distinguishes these breast cancer subtypes. Interestingly, differential isoform expression alone was sufficient to distinguish ER+ and TN breast cancer subtypes, indicating that promoter usage, splicing, and 3’UTR usage may be differentially regulated in breast cancer subtypes. We replicated these findings in more than 600 cases from two additional cohorts and using an independent technology for assaying RNA isoform expression. Finally, we identified differentially expressed RNA processing factors that are responsible for subtype-specific splicing, and we demonstrated that RNAi knockdown of these factors affects subtype-specific isoform expression levels in an ER+ breast cancer cell line.

## Results

### Isoform usage differentiates breast cancer subtypes

**Fig 1:**
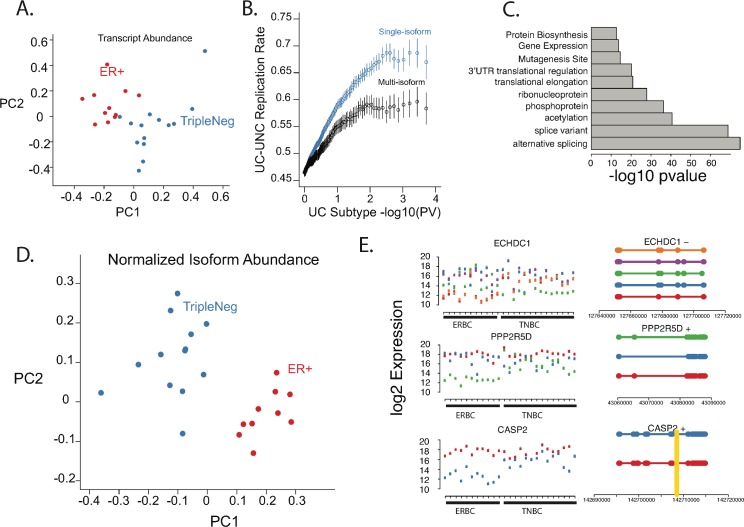
Breast Cancer Isoform Abundance differentiates breast cancer subtypes. A) First two principal components derived from RefSeq gene RNAseq FPKM expression levels. As expected, expression levels can differentiate breast cancer subtypes. B). Replication rate between microarray and RNAseq for subtype-specific, differentially expressed genes comparing single isoform and multi-isoform genes. The replication rate is higher for single isoform rather than multi isoform genes. C) Top 10 enriched pathways from DAVID analysis on differentially expressed genes between subtypes (plotting–log10 p value). Genes with multiple isoforms are highly enriched. D) First two principal components derived from RefSeq gene RNAseq FPKM expression levels for multi-isoform genes only. Differential expression of isoforms alone is sufficient to segregate breast cancer subtypes E) Left panel, by sample FPKM expression levels for ECHDC1, PPP2R5D, and CASP2. Each color represents a specific isoform. Right Panel, Isoform gene models, colors corresponding to expression plots. Thick bars = exons, thin bars = introns. Vertical gold bars = ESR1 binding site from ChIPseq data.

RNA-sequencing allows accurate assessment of transcript abundance, identification and quantification of isoform usage and efficient discovery of fusion-genes [17,18]. To elucidate differences in these processes between ER+ and TN subtypes, we sequenced the transcriptomes of 11 ER+ and 14 TN from frozen specimens obtained from the University of Chicago pathology core, using paired-end Illumina sequencing (S1 Table). We aligned reads to the human reference genome using a splice-junction aware aligner (Tophat) [19,20] (S2 Table). Expression levels were estimated for 32,041 RefSeq transcripts, corresponding to 22,996 non-overlapping gene models (S3 Table), expressed in at least two tumors. ER+ and TN breast cancers are known to differ in their expression profiles [8]. Quantitative analysis of fragments per kilobase per million mapped reads (FPKM) revealed 7,415 RefSeq transcripts that have subtype-specific expression patterns (Wilcox Rank Sum test, false discovery rate < 0.05, S3 Table). In accordance with previous observations [8], tumor subtypes were clearly distinguishable on the basis of expression level (Fig 1A). Subtype-specific gene expression levels were largely replicated between our RNAseq data and previously published microarray data sets from independent cohorts [21](S1 Fig).

As microarray based differential expression measurements may be confounded by isoform specific microarray probes, we explored the replication rate between microarray data and RNAseq data for single isoform and multi-isoform Refseq genes. As expected, we observed a higher replication rate for single isoform genes as opposed to multi-isoform genes (Fig 1B). We next explored the function of differentially expressed genes in our dataset, using pathway analysis and Gene Set Enrichment Analysis (GSEA) [22,23]. Reassuringly, the top gene sets identified by GSEA in both TN and ER+ tumors are those that distinguish breast cancer subtypes in previous studies (S2 Fig). Indeed, the gene sets identified by GSEA were dominated by breast cancer expression sets. This analysis also highlighted several interesting differences in the biology between ER positive and triple negative breast carcinomas. For example, gene sets involved in the biogenesis of peroxisomes were enriched in ER+ breast cancer (S4 Table). Interestingly, it has been shown that the pentose phosphate shunt, which depends on peroxisomes, is particularly active in ER+ breast cancer cell lines and its activity is dependent upon estrogen signaling [24]. Conversely, GSEA identified several cytokine and immune related gene sets that are upregulated in triple negative breast cancers; these pathways likely reflect the prominent immune infiltrates common within triple negative tumors and that are apparent by morphology (S4 Table).

We also performed DAVID analysis of differentially expressed genes, and this approach demonstrated enrichment for regulatory processes that affect both gene and protein expression levels and function, such as phosphoproteins, 3’-UTR mediated translational regulation and gene expression (Fig 1C). The most significant biological process enriched in our differentially expressed gene set was alternative splicing (Fig 1C). Identification of alternative splicing was particularly intriguing given the ability of transcriptome sequencing to systematically quantify isoform expression levels [17,18]. Indeed, differentially spliced isoforms are emerging as an important factor in carcinogenesis [9,10,25]. Interestingly, differential isoform usage alone was sufficient to distinguish ER+ and TN subtypes (Fig 1D). To further explore this finding, we analyzed the expression of 5,408 Refseq gene models with more than one isoform. 694 of these genes exhibit subtype-specific isoform expression levels (subtype by isoform interaction using an ANOVA F-test, false discovery rate < 0.05; Fig 1E, S3 Table). This result indicates that alternative promoter usage, alternative splicing, and alternative 3’UTR usage are differentially regulated between the subtypes and may contribute to the differing biology of ER+ and TN breast cancer subtypes.

Fig 1E shows several examples of differential isoform expression between ER+ and TN tumors. ECHDC1, an enzyme involved in mitochondrial fatty acid metabolism and located in a region implicated in breast cancer by GWAS [26], showed differential splicing between ER+ and TN subtypes (Fig 1E; ANOVA F-test, FDR = 7.95e-08). PPP2R5D is a regulatory subunit of protein phosphatase 2A, which is implicated in the negative control of cell growth and division [27]. There are three isoforms of PPP2R5D that encode different proteins, and we found that one of the short isoforms is predominantly expressed in TN tumors (Fig 1E; ANOVA F-test, FDR = 8.067e-07). Caspase 2, which mediates the execution phase of apoptosis, is known to express 2 isoforms that encode different proteins; the shorter isoform is believed to protect from apoptosis, while the longer isoform promotes apoptosis [28,29]. We found the short, protective isoform of CASP2 to be predominantly expressed in TN tumors (Fig 1E; ANOVA F-test, FDR = 2.34e-05), suggesting a potential mechanism that TN tumors could use to prevent apoptosis. Interestingly, GSEA of differentially expressed isoforms demonstrated enrichment for genes involved in apoptosis, and thus differential splicing may contribute more broadly than CASP2 to differences in regulation of apoptosis between breast cancer subtypes (S3 Fig).

### Replication of isoform usage discrimination of breast cancer subtypes

To replicate subtype-specific isoform usage, we used data generated by The Cancer Genome Atlas (TCGA).

**Fig 2:**
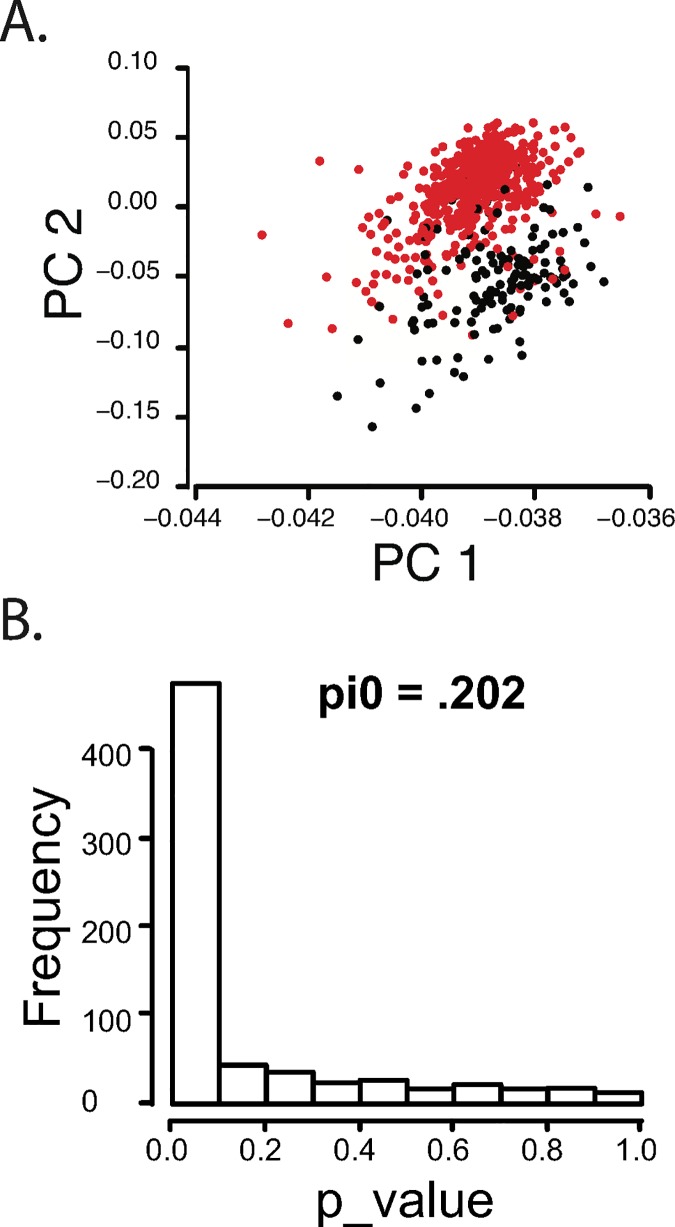
Breast Cancer Isoform Abundance Differentiates Breast Cancer Subtypes in TCGA data. A) First two principal components derived from RefSeq gene RNAseq FPKM expression levels for multi-isoform genes only. TCGA samples are segregated by breast cancer subtype. B) P value plot for subtype-specific isoform expression in the TCGA data for 694 multi-isoform genes that were differentially expressed in the discovery cohort. 80% of these genes also show subtype-specific isoform expression in the TCGA cohort.

We downloaded and aligned 594 breast cancer RNAseq data sets from TCGA, following the same data processing pipeline used for our internal dataset [30]. As in our discovery cohort, principal component analysis demonstrated that ER+ and triple negative tumors could be differentiated by isoform usage alone (Fig 2A). Expression of single isoform only genes was also able to differentiate ER+ and TN breast cancer subtypes, consistent with previous observations that gene expression differs strongly between breast cancer subtypes (S4 Fig). Thus, not only can breast cancer subtypes be differentiated by differential expression of different genes, they can also be differentiated by isoform usage alone. In our discovery dataset, we identified 694 multi-isoform genes with subtype-specific differential isoform expression. Analysis of these same 694 multi-isoform genes in the TCGA dataset demonstrates that the vast majority replicate significant subtype-specific isoform expression (Fig 2B).

**Fig 3:**
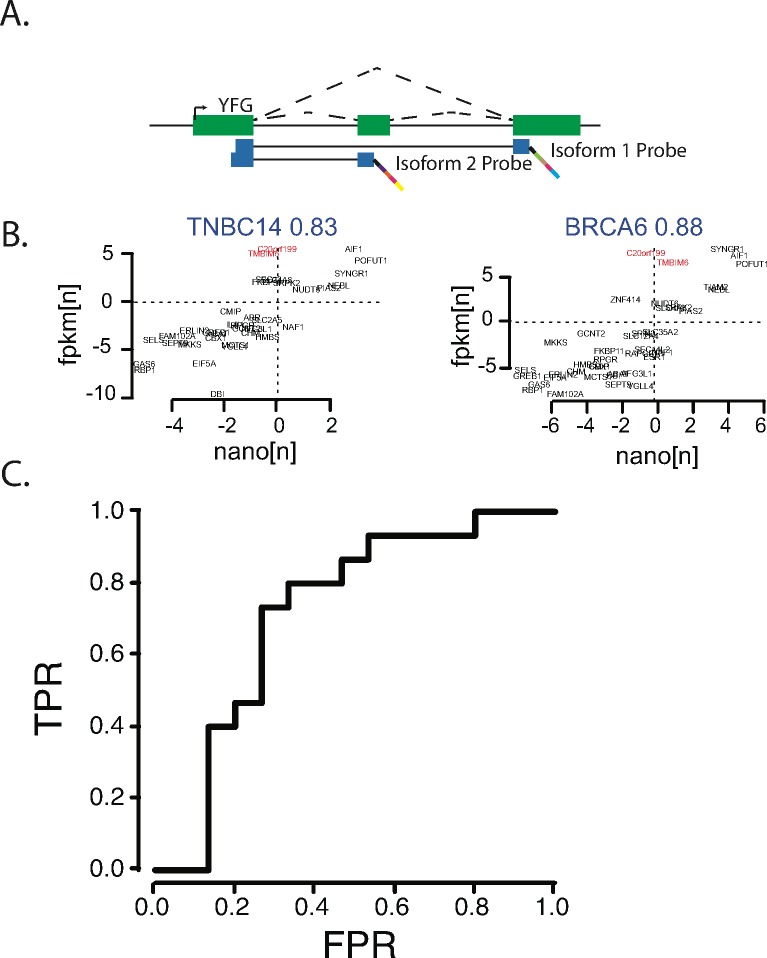
Nanostring expression isoform expression levels replicate ability to discriminate breast cancer subtypes in additional cohort. A) Schematic of isoform specific nanostring probes. B) In the discovery cohort, RNAseq isoform expression ratios versus nanostring isoform expression ratios, demonstrating significant concordance between the two platforms C) Receiver-operator curve for logistic model trained on nanostring isoform expression levels in the discovery cohort and applied to a validation cohort of 64 breast cancer cases. (AUC = .76)

To further validate our results, we designed isoform-specific Nanostring probes for 212 isoform pairs that showed differential subtype expression in the original 25 samples (Fig 3A). To assess technical validation of our isoform measurements, the expression log ratio of each isoform pair was calculated for each platform; concordant isoforms are represented in the upper right and lower left quadrants (example in Fig 3B, S5 Fig). Each of our samples showed between 80–85% concordance between Nanostring and RNAseq data, indicating that subtype-specific differential isoform usage was largely reproducible. Furthermore, many of the discordant probes were discordant across the majority of the samples, suggesting that the observed non-concordance between RNAseq and Nanostring was typically due to a small subset of isoforms that were consistently, inaccurately measured on a particular platform.

To determine if the ability of differential isoform expression to segregate ER+ and TN subtype was biologically reproducible, we measured isoform expression via Nanostring in an independent set of 68 breast cancer specimens obtained from FFPE blocks from the University of Chicago pathology core (44 ER+ and 24 TN). To assess the ability of our isoform expression signature to distinguish ER+ and TN breast cancer subtypes in this independent cohort, we used the Nanostring data from the discovery cohort to build a logistic regression-based subtype classifier and applied that classifier to the replication cohort. Subtype prediction based on isoform expression was consistent using this replication cohort and an independent method for RNA measurement (Fig 3C, AUC = 0.72), further demonstrating that ER+ and TN breast cancer subtypes can be differentiated based on isoform expression alone.

### Splicing, alternative promoter usage and alternative 3’UTR usage contributes to differences between breast cancer subtypes

**Fig 4:**
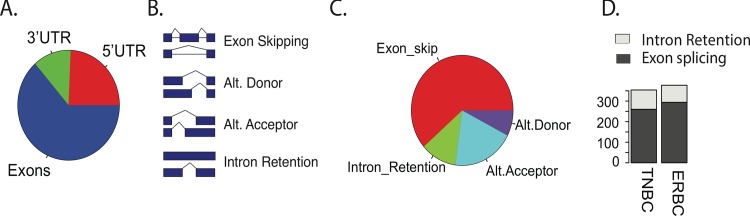
Differential isoform usage between breast cancer subtypes includes alternative splicing, alternative promoter usage, and alternative 3'UTR usage. A) Fraction of differentially expressed isoforms that differ in exon usage, 3’UTR usage, or 5’UTR usage, pairwise comparison. No one mechanism dominates. B) Schematic outlining different types of splicing, including exon skipping, intron inclusion, and alternative donor/acceptor sites. C) Pairwise comparison of exon splicing events shows fraction of each type occurring within differentially expressed isoforms. D) Plot of the number of exon skipping and intron retention events in each subtype. There is no significant difference between subtypes.

In the analysis above, we identified 694 multi-isoform genes that showed subtype-specific differential expression. To understand the mechanisms that generate subtype-specific isoform usage, we quantified subtype-specific expression for all possible pairs of isoforms of the 694 genes. Differentially expressed isoform pairs (ANOVA F-test, FDR < 0.05) were then compared to determine whether they differed in 5’UTR, exons, and/or 3’UTR. 967 isoform pairs were found to be differentially expressed. The majority of differentially expressed pairs were alternatively spliced (63.5%), but differential promoter usage (24.3%) and differential 3’UTR usage (12.2%) were also common (Fig 4A). These fractions were not significantly different than the distribution of isoform pairs in RefSeq, indicating that no single mechanism predominates in differentiation of ER+ and TN breast cancer subtypes. Considering the largest category, alternatively spliced isoforms, the difference between isoforms can be defined as exon skipping events, intron retention events, alternative donor events, or alternative acceptor events (Fig 4B). To determine the fraction of each event, we counted all such events for all pairwise, differentially expressed isoforms. Exon skipping events represented the majority, accounting for 61.4% of alternate splicing events, while intron retention, alternative acceptor and alternative donor accounted for 11.4%, 19.6%, and 7.6%, respectively (Fig 4C). We then determined if exon splicing or intron retention predominated in one of the breast cancer subtypes, or if such events were equally distributed between subtypes. For each differentially expressed pair we determined whether the isoform with the skipped exon or retained intron was more highly expressed in TN or ER+ tumors. There was no significant difference between ER+ and TN subtypes in number of expressed isoforms with retained introns or skipped exons, indicating that the differences in splicing between subtypes is due to selection of target genes for splicing, rather than general predominance of a particular general splicing mechanism in one subtype (Fig 4D).

We next examined the role that differential 5’UTR and 3’UTR usage played in differentiating ER+ and TN subtypes. Alternate UTR usage could result in either change of coding sequence or of only non-coding sequence. For each pair of differentially expressed isoforms that differed in UTR sequence, we determined whether the resulting differences also resulted in coding sequence differences. 50% of all 5’UTR differences resulted in coding sequence differences between isoforms, while the vast majority of 3’UTR differences were accompanied by changes in coding sequence (S6 Fig)

### Differential expression of RNA processing factors between breast cancer subtypes contributes to isoform usage differences

**Fig 5:**
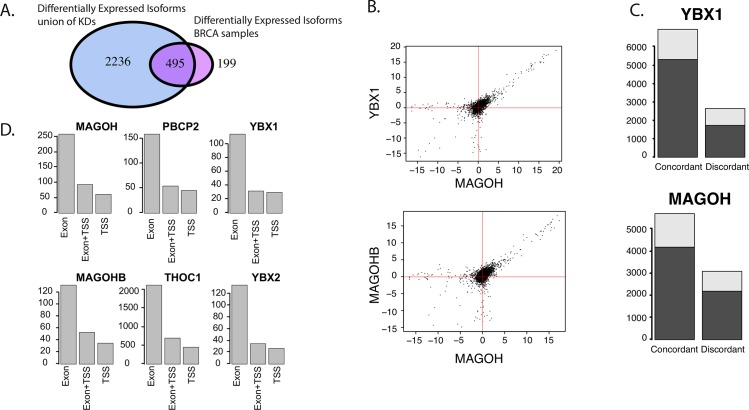
Knockdown of RNA processing factors differentially expressed between subtypes alters expression of subtype-specific isoforms. A) Venn diagram showing the overlap for isoforms affected by splicing and isoforms differentially expressed between subtypes in our discovery cohort. In the case of both MAGOH and YBX1, the overlap is significant. (Fisher’s Exact Test). B). Top panel: Plot FPKM of multi-isoform genes from MAGOH knockdown (x-axis) and YBX1 knockdown (y-axis). Bottom panel: Plot FPKM of multi-isoform genes from MAGOH knockdown (x-axis) and MAGOHB knockdown (y-axis). In both cases, there is a high degree of overlap in isoform expression levels. C) Isoforms were classified into concordant or discordant based on direction of changes in the knock-down compared to expected direction of change from the breast cancer sequencing data. Many more isoforms were concordant than non-concordant, and there was no difference in fraction of concordant genes whether they were up regulated by knockdown (dark grey) or down regulated by knockdown (light gray) D) Pairwise differentially expressed isoforms (FDR <0.05) are mostly represented by isoforms that differ in exons, rather than TSS, indicating that knockdown of these RNA processing factors is affecting splicing.

We hypothesized that differential expression of RNA processing factors generates these differences in isoform expression. Using a list of human RNA processing factors derived from Uniprot (S5 Table), we found that 57 of 194 RNA processing factors were differentially expressed between the subtypes, and thus splicing factors were enriched in differentially expressed genes (Fisher test p.value = 8.38e-5). Given that ER expression contributes to, and to some extent defines, the differences between ER+ and TN breast cancer subtypes, we further hypothesized that splicing factors directly or indirectly regulated by estrogen receptor were likely to contribute to subtype-specific differential splicing. Thus, to test this hypothesis and to identify determinants of differential splicing between ER+ and TN breast cancer subtypes, we selected several RNA processing factors that were differentially expressed between breast cancer subtypes (S7 Fig, S6 Table), were induced by estrogen in an estrogen time course in MCF7 cells[31], and/or have previously been identified as potential direct targets of ER based on the presence of ER binding sites in genome-wide chromatin immunoprecipitation experiments[31]. Because we wished to use ER+ MCF7 cells to carry out functional validation of candidate RNA processing factors, we also required that the factors we selected to be expressed at detectable levels in MCF7 cells. The resulting set of RNA processing factors included MAGOH, MAGOHB, YBX1, YBX2, THOC1, and PCBP2. MAGOH and MAGOHB are closely related members of the same family and both are known to function in the exon junction complex. MAGOH was strongly differentially expressed between tumor ER+ and TN subtypes, while MAGOHB was regulated by estrogen in MCF7 cells. MAGOH, MAGOHB, YBX1, PCBP2, and to a lesser extent THOC1, showed strong subtype-specific expression in the TCGA dataset (S8 Fig, S6 Table), while YBX2 showed strong estrogen regulation in the MCF7 time course. Each of these factors was knocked down via siRNA in MCF7 cells, and gene expression was assayed via RNAseq to determine what effect knockdown had on isoform usage (S9 Fig). For each knockdown, differentially expressed isoforms were defined by comparison to control, and then the overlap of these differentially expressed isoforms was compared to differentially expressed isoforms in the RNAseq tumor expression data. This overlap was significant for all 6 RNA processing factors (Fig 5A, S10 Fig, p-values in Table 1). Indeed, of the 694 multi-isoform genes that showed subtype-specific expression in our discovery cohort, 495 showed isoform specific changes in expression with knockdown of at least one of the chosen processing factors (Fig 5A). Interestingly, the overlap of differentially expressed isoforms upon knockdown of each of the six RNA processing factors was also highly significant, suggesting a unified splicing program mediated by these factors differentiating ER+ and TN breast cancer subtypes (Fig 5B, Table 1). Indeed, pairwise comparison of gene expression levels between splicing factor knockdowns showed a high degree of correlation for all pairs of RNA processing factors (Fig 5B and 5C, Table 1). Importantly, the direction of change of isoform expression was largely concordant between the knock-down and the expected direction of change from the breast cancer sequencing data (Fig 5C). We next asked whether the isoforms that are differentially expressed differ in exons, UTRs, or both. Fig 5 panel D demonstrates that differential exon utilization accounts for the vast majority of differentially expressed isoforms following knockdown of all 6 factors. This result is consistent with knockdown of these factors affecting splicing.

**Table 1:** Isoform Overlaps between Knockdown and Tumor samples.

	Gene	MAGOH	MAGOHB	PCBP2	THOC1	YBX1	YBX2
Subtype vs knock down	subtype_and_KD	63	29	47	403	30	31
	subtype_not_KD	606	609	611	551	611	611
	KD_not_subtype	275	161	183	2133	130	143
	not_KD_not_subtype	4243	4256	4254	4005	4260	4260
	Odd ratio (95% CI)	1.6 (1.18–2.15)	1.26 (0.81–1.9)	1.79 (1.25–2.51)	1.37 (1.03–2.43)	1.61 (1.03–2.43)	1.51 (0.98–2.26)
	Fisher Extact Test p-Value	0.0018	0.2652	0.0012	9.20E-06	0.0291	0.0483
ER binding vs knock down	ER_and_KD	153	72	65	1860	69	56
	ER_not_KD	6695	6776	6783	4988	6779	6792
	KD_not_ER	568	270	314	6835	216	194
	not_KD_not_ER	29338	29636	29592	23071	29690	29712
	Odd ratio	1.18 (0.98–1.42)	1.17 (0.89–1.52)	0.9 (0.68–1.18)	1.26 (0.92–1.71)	1.4 (1.05–1.85)	1.26 (0.92–1.71)
	Fisher Extact Test p-Value	0.07	0.26	0.51	7.69E-14	0.018	0.14
PR binding vs knock down	PR_and_KD	68	34	32	688	29	21
	PR_not_KD	2387	2421	2423	1767	2426	2434
	KD_not_PR	653	308	347	8007	256	229
	not_KD_not_PR	33646	33991	33952	26292	34043	34070
	Odd ratio	1.47 (1.12–1.89)	1.55 (1.05–2.22)	1.29 (0.87–1.86)	1.28 (1.16–1.4)	1.59 (1.04–2.34)	1.28 (0.78–2.01)
	Fisher Extact Test p-Value	0.004	0.021	0.178	2.37E-07	0.023	0.253
Subtype expression vs knock down	knockdownUp_tnDown	4186	5390	4111	3177	5321	4292
	knockdownDown_tnDown	2192	1430	1572	2978	1738	1908
	knockdownUp_tnUp	1515	1658	1232	1084	1642	1466
	knockdownDown_tnUp	898	814	798	1003	913	802
	Odd ratio	1.13 (1.03–1.25)	1.85 (1.67–2.05)	1.69 (1.52–1.89)	0.99 (0.89–1.09)	1.7 (1.54–1.88)	1.23 (1.11–1.23)
	Fisher Extact Test p-Value	0.013	2.20E-31	7.10E-22	0.8	3.06E-26	7.09E-05
Splicing factor subtype expression	SF_expresion_TN-ER	0.862	0.225	-0.106	-0.155	1.32	0.687

We next asked whether splicing targets of these factors were also known ER and PR targets, using previously published ChIP binding data[31]. Interestingly, targets of THOC1 and YBX1 show enrichment for ER and PR binding sites, while targets of MAGOH and MAGOHB show enrichment for ER binding sites (Table 1, S11 Fig, S12 Fig). Most significantly, for all the RNA processing factors except THOC1, the direction of the effect on isoform expression is consistent between the knockdown and expectation based on the isoform profiles of primary tumors (Table 1). These results indicate that differential isoform usage between breast cancer ER+ and TN subtypes is regulated by differential expression of several RNA processing factors, including MAGOH, MAGOHB, YBX1, YBX2, and PCBP2. Thus, we have identified a splicing driven signature that differentiates breast cancer subtypes and identified RNA processing factors that contribute to subtype differences.

## Discussion

A better understanding of the biological mechanisms that underlie gene expression differences between ER+ and TN breast cancer subtypes promises to yield new insights into subtype-specific therapeutic targets. Here we have used paired-end, whole transcriptome sequencing to identify a set of isoforms that distinguish ER+ and TN tumors. Recently published studies have strongly implicated alternative splicing as an emerging factor in tumorigenesis [9,10][11,12]. A previous RNA sequencing study established that splicing is dysregulated in breast cancer compared to normal controls, and that the genes that are differentially spliced between normal and cancer vary based on subtype[32]. In our studies, we demonstrate that not only does splicing differ between breast cancer subtypes, but also quantitative assessment of differential splicing alone was sufficient to distinguish ER+ from TN tumors.

Our finding that differential transcript isoform usage can differentiate breast cancer subtypes is strongly reproducible. Using only ~200 isoforms, we demonstrate this finding in three independent cohorts, University of Chicago Frozen, University of Chicago FFPE, and TCGA. Thus, breast cancer subtypes could be distinguished based on the expression of different versions of the same genes. These results suggest that subtype-specific, differential regulation of promoter usage, alternative splicing, and 3’UTR usage contributes to differences in gene expression and thus differences in biological behavior between subtypes. We find that many RNA processing factors are differentially expressed between ER+ and TN breast cancer subtypes. Furthermore, we have identified several RNA processing factors, including YBX1, YBX2, MAGOH, MAGOHB, and PCBP2, that appear to contribute to subtype-specific splicing. We have shown that, not only are these RNA processing factors differentially expressed between subtypes, but also that when they are knocked down, splicing changes in the direction predicted. In other words, knockdown of factors expressed in ER+ BRCA led to a more TN-like splicing pattern, and vice versa. Thus, global dysregulation of splicing events is a fundamental component of the breast cancer transcriptome that occurs in a subtype-specific and reproducible manner. Differential expression of splicing factors between ER+ and TN breast cancer subtypes alters the transcriptome in predictable ways that likely contribute to differences in biology between breast cancer subtypes. Addition of splice usage to breast cancer transcriptome studies may further refine our ability to distinguish breast cancer subtypes, as well as perhaps identify fine details within subtypes. Of note, we also attempted to knock-down these factors in a MBA-MD-468 and measure isoform usage, but the knock-down was lethal to these cells.

Additionally, further study of the splicesome may lead to improved understanding of breast cancer biology and suggest several ways in which altered splicing may affect phenotype in breast cancer subtypes. For example, TN tumors collectively express a short, anti-apoptotic isoform of caspase 2 that was not expressed in ER+ tumors[28,29]. Thus, TN tumors may use splicing of caspase 2 in avoid apoptosis in a way that ER+ tumors do not. This finding could have therapeutic implications; such splicing may make one of the subtypes more or less susceptible to therapies designed to promote apoptosis. Conversely, if this splicing event could be blocked in TN tumors, it could induce apoptosis. Future work will focus on identification of differentially spliced isoforms that explain differences in biological behaviors between subtypes.

Thus, in this work, we have used RNA-seq to mine the transcriptome of two common invasive breast cancer subtypes; ER+ and triple negative breast cancer. We demonstrate that, in addition to transcript abundance, isoform abundance is sufficient to distinguish breast cancer subtypes, and implicate differences in isoform usage as underlying some of the differences seen in the biology of these two tumors. We have identified differentially expressed RNA processing factors, and demonstrate that they are responsible for splicing of subtype-specific isoforms.

## Methods

### Sample procurement

Twenty-five frozen breast carcinoma samples (11 ER positive and 14 triple negative) were obtained from the de-identified tissue bank in the Human Tissue Resource Center in the Department of Pathology at the University of Chicago Medical Center. A de-identified pathology report was supplied with each case, which contained grade, stage, lymph node status, age +/- 5 years and self-reported race (S1 Table). Additionally, a frozen section from the block that was used for RNA isolation was provided with each case. These sections were examined by a pathologist to confirm the diagnosis and grade, as well as to ensure each block contained at least 70% tumor. These slides were also used to select areas of high tumor concentration for RNA isolation (see below). Slides were also cut for adjacent normal to confirm the absence of tumor. Additionally, 68 de-identified FFPE samples, representing 44 ER+ and 24 ER- samples were obtained from the diagnostic archives of the Department of Pathology at the University of Chicago Medical Center. This study was approved by The University of Chicago Institutional Review Board (protocol 16970B), and waiver of consent was granted as no identifying personal health information was provided to researchers.

### Library preparation and sequencing

cDNA libraries were prepared for Illumina sequencing with minor modifications to published protocols(*36*, *37*). Briefly, frozen section slides cut from each block were used to select areas with high tumor cell concentration. These areas of the frozen block were then punched using a sharpened 1mm tissue microarray punch. OCT was removed from the ends of this punch via a scalpel, and the frozen core was ground to a fine powder under liquid nitrogen. Total RNA and gDNA was then extracted from frozen tissue samples using Qiagen QiaShredder/RNeasy and DNeasy Blood and Tissue kits. RNA quality was confirmed using an Agilent Bioanalyzer, and no RNA with a RIN < 7.5 was used. Poly-adenylated RNA was purified with the micropolyA purist kit (Ambion), ethanol precipitated, and resuspended in 10 uL RNAse free water. First strand reverse transcription was primed with random nonomers, using SuperscriptII RT (Invitrogen). Second strand synthesis was performed with RNAse H and DNA Polymerase I. Double stranded cDNA was repaired and polished with T4 DNA polymerase, Klenow, and T4 polynucleotide kinase. Adenosine was added to 3' ends with Klenow and paired end Illumina adapters ligated with T4 DNA ligase. Ligation reactions were size selected for 400–500 bp fragments after agarose gel electrophoresis and PCR amplified for 15 cycles using Illumina PCR primers and Pfx polymerase. Paired end Illumina sequencing was performed according to the manufacturer's instructions. RNA from MCF7 knockdowns was isolated using Qiagen RNeasy kits, and RNAseq libraries were built using ScriptSeq kits from Epicentre, following manufacturer’s protocols.

### Read mapping

Illumina sequence files were converted to Sanger fastq format (ie., Q0-93 using ASCII 33–126). Paired end reads were aligned to the human reference genome (build 36) using TopHat. Tophat was run with an estimated mean and standard deviation insert size of 200 and 50 bases, respectively. Sequencing artifacts occasionally produced lanes with fewer than 20% aligned reads. Raw data QC revealed that many such cases were caused by precipitous drops in sequence quality at late run cycles. In such cases, reads were trimmed from the 3' end such that, on average across all reads in a lane, the Phred quality score based estimate of the probability of a sequencing error within the remaining read was less than 95%. TopHat alignments were generated in SAM format(*39*) and further alignment manipulation was performed with SAMTools, Picard (http://picard.sourceforge.net), and custom perl scripts. Read alignments with mapping qualities less than 10 were removed. Alignments generated from more than one sequencing lane were merged with Picard MergeSamFiles. We downloaded TCGA consortium aligned RNA-seq bams for 657 samples from cgHub (https://cghub.ucsc.edu, TCGA's Data Coordinating Center) as a member of the TCGA working group. TCGA's Breast RNA-seq libraries are generated using the Illumina TrueSeq library preparation protocol and sequenced on the Illumina HiSeq 2000 machines. A median of 76.4 million 2x50bp reads were generated for each sample. Using in-house scripts, we generated raw fastq sequence files from the bams for re-analysis.

We re-aligned all fastqs and generated gene/isoform quantification using the tophat and cufflinks, respectively. To allow for proper comparisons, all parameters, software versions, reference genomes and gene models used for this analysis are consistent with the pipeline we used to analyze the original 26 samples in this study. Briefly, we trimmed poor quality bases (to Q15) at the 3' ends of the reads using a trimming algorithm implemented in BWA. Tophat requires the parameters for insert size distribution of the RNA-seq library. We determined for each sample this by sub-sampling 1 million reads and re-aligning them to the spliced transcriptome using bowtie-2.0.0-beta. Mean and standard deviation are determined from the reads that align concordantly to the transcriptome using the CollectInsertSizeMetrics module in Picard suite of tools. We next aligned all reads to hg18 reference genome using tophat-1.3.3 (—no-coverage-search—segment-length 25—segment-mismatches 2). We also provided the gene models to the aligner at this step for faster and accurate resolution of known splice junctions. Transcript quantification on the aligned bams is performed using cufflinks-2.0.0 (all default parameters).

### Gene expression analyses

RNASeq: Transcript expression levels were estimated directly from each lane of RNAseq data, as Fragments Per Kilobase per Million mapped reads (FPKM) using cufflinks (http://cufflinks.cbcb.umd.edu/) for transcript assembly and quantitation, with RefSeq gene models as a reference annotation set. FPKM distributions were quantile normalized across lanes. For principal component analysis (performed via pcaMethods package in R), FPKMs were mean centered. Subtype-specific differential gene expression was calculated in two ways 1) via gene-by–gene linear model, of the form y ~ m0 + subtype + E and 2) by Wilcoxon rank sum test, comparing the two subtypes. Subtype-specific isoform expression was calculated via gene gene-by–gene linear model, of the form y ~ m0 + subtype + isoform + subtype*isoform + E, where the interaction between isoform and subtype identified differentially expressed genes. All effects were treated as fixed effects, and multiple hypothesis testing was controlled via Storey’s q-value FDR.

### Microarray

Microarray expression data from GSE10866 was downloaded from Gene Expression Omnibus into R using GEOquery. The expression set was subset to arrays using the GPL1390 Agilent Human 1A Oligo UNC custom microarray. Subtype-specific differential gene expression was calculated via gene-by–gene linear model (R function lm), of the form y ~ m0 + subtype + E. The intensities of probes targeting the same gene were averaged. Tumor subtype was treated as a fixed effect, significance of the tumor subtype effect was assessed by t-test, and multiple hypothesis testing was controlled via Storey’s q-value FDR. Estrogen time course data was analyzed as in Hua et. al.[31]

### Nanostring

Isoforms with significant subtype*isoform interactions (FDR = 0.05) were submitted to Nanostring for custom design. Two hundred and twelve isoforms, representing 106 genes, could be designed with high quality nanostring probes unique for each isoform. A codeset specific to the targets was designed using a 3’ biotinylated capture probe and a 5’ reporter probe tagged with a specific fluorescent barcode; creating two sequence-specific probes for each target transcript. Probes were hybridized to 100 ng of total RNA for 19 hours at 65°C. Following incubation, samples were applied to the NanoString Preparation Station for automated removal of excess probe and immobilization of probe-transcript complexes on a streptavidin-coated cartridge. All manipulations were performed using the NanoString Preparation Station robotic fluids handling platform. Data were collected using the nCounter^TM^ Digital Analyzer by counting the individual specific fluorescent barcodes and quantification of target RNA molecules in each sample.

### Nanostring data processing and class prediction analysis

All normalization and analysis of the Nanostring data was performed in R. Nanostring data were normalized by fitting a negative binomial model with terms for assay, well, median negative control, median house keeping gene, and transcript. The resulting residuals were used for subsequent analysis. To evaluate concordance between RNAseq and nanostring expression levels, the ratio of each isoform pair was calculated for all samples in which RNAseq and nanostring data was obtained. To determine the ability of isoform expression to differentiate subtypes, the nanostring data from the 26 samples in our discovery cohort was used to train a logisitic regression classifier. The weights from this regression classifier were then extracted and used to classify each of the 68 FFPE samples into either ER+ or ER- cases. This classification was compared to the pathologic diagnosis, and the performance of the classifier assessed by receiver-operator characteristics.

### Isoform analysis

AStalavista was used to extract splicing events from refseq gene models. For all gene symbols with multiple refseq isoforms, subtype-specific isoform expression for all possible isoform pairs was calculated via gene gene-by–gene linear model, of the form y ~ m0 + subtype + isoform + subtype*isoform + E, where the interaction between isoform and subtype identified differentially expressed genes. By comparing isoforms in a pairwise fashion, we could determine whether subtype-specific isoforms differ in 5’UTR, 3’UTR or coding sequence. For differentially expressed isoforms that differ in 5’UTR sequence, 1000bp upstream of each different 5’UTR sequence was extracted. Beta coefficients from the linear model were used to determine which isoform was expressed in each subtype to allow separation of ER+ 5’UTR and TNBC 5’UTRs. These sequences were supplied to MEME 2.6 to identify enrichment of transcription factor binding sites.

### Knockdown expression analysis

Illumina reads were treated as above, and aligned to hg18 with Tophat. Cufflinks with refseq gene models was used to assemble transcripts. Cuffdiff was used to identify differentially expressed isoforms, using refseq genes models as a reference.

### Cell culture/siRNA

MCF7 were cultured in DMEM/10% FBS + penicillin/streptomycin. Dharmacon ON-Targetplus SMARTpools were ordered for each of the splicing factors, plus negative controls. siRNA transfection used DharamaFECT1 and followed manufacturer’s protocols. Briefly, cells were trypsanized, counted and diluted to 1x10^5 cells/ml, and 3 mls were plated/well in 6-well plates. Cells were incubated at 37C overnight. Cells were transfected the next day with 2uM siRNA using DharmaFECT1. RNA was harvested using Qiagen RNeasy, following manufacturer’s protocols, 72 hours after transfection. Experiments were performed as biological duplicates. Knockdown was confirmed using qrtPCR, using one step RT-PCR SYBR Green master mix from BioRad, following manufacturer’s instructions. qPCR was performed in an ABI StepOne real time PCR machines. GAPDH primers were used as a control. All primer sequences are shown in Table 2.

**Table 2:** Primers for qPCR.

MAGOHB	F	TTG GCC GAC AGG AGC TTG AAA TTG
	R	AGG CCT TCA GGA TCC TTT GAC TGA
PCBP2	F	CAC CAG TTG GCA ATG CAA CAG TCT
	R	ATG CAT CCA AAC CTG CCC AAT AGC
THOC1	F	ACA AGG GAA CAC ATG CCC ACT TTG
	R	AAG TGA GGG CTT CTC CGT GCT AAT
YBX2	F	ACG TCC GGA ATG GTT ACG GAT TCA
	R	TAC ATT AGT GGC TTC TGC GCC CTT
MAGOH	F	TCT TGG AAG TTC AGG CTC GGT TGT
	R	ATC TTA ACT TCC CGT CCG GTC GAA
YBX1	F	AGG TCA TCG CAA CGA AGG TT
	R	TGC ACA GGA GGG TTG GAA TAC TGT
GAPDH	F	TCG ACA GTC AGC CGC ATC TTC TTT
	R	ACC AAA TCC GTT GAC TCC GAC CTT

## Supporting information

S1 Fig RNAseq expression data recapitulates microarray expression data. Plot of the beta coefficient from the linear model used to identify subtype-specific expressed genes, with RNAseq data plotted on the x-axis and microarray data plotted on the y-axis. RNAseq is highly concordant with microarray data. ESR1, FOXA1, and XBP1 (in red), which are known to be highly differentially expressed between breast cancer subtypes, are highly differentially expressed in both RNAseq and microarray data sets.
(TIF)

S2 Fig Subtype-specific differentially expressed genes from RNAseq data recapitulate previous data sets. Top two gene sets from Gene Set Enrichment Analysis of normalized RNAseq data are genes up-regulated and down-regulated in ER+ breast cancer.
(TIF)

S3 Fig Subtype-specific isoforms are enriched for genes in the apoptotic pathway. A) Top 10 enriched pathways from DAVID analysis on differentially expressed isoforms between subtypes (plotting–log10 p value). Genes in apoptotic categories are enriched. B) Top gene sets from Gene Set Enrichment Analysis of Isoform FPKM data show enrichment for apoptotic gene modules.
(TIF)

S4 Fig Single isoform genes alone differentiate breast cancer subtypes. First two principal components derived from RefSeq gene RNAseq FPKM expression levels for single isoform genes only. TCGA samples are segregated by breast cancer subtype.
(TIF)

S5 Fig Nanostring isoform expression validates RNAseq isoform detection. Each panel is represents one of the initial discovery samples, with the ratio of two isoform expression levels from RNAseq data plotted on the x-axis and the ratio of two isoform expression levels from nanostring data plotted on the y-axis.
(TIF)

S6 Fig Subtype-specific isoforms that differ at the 5’ and 3’ UTR include isoforms that differ in coding sequence and isoforms that differ only in noncoding sequence. There is no significant difference between subtypes.
(TIF)

S7 Fig MAGOH and YBX1 are differentially expressed between breast cancer subtypes. Left panel shows FPKM expression level, by sample, for MAGOH (top) and YBX1 (bottom). Right panel show the gene model for MAGOH (top) and YBX1 (bottom), with genomic position on the x-axis, exons represented as thick lines, and introns represented as thin lines.
(TIF)

S8 Fig Splicing factors are differentially expressed between breast cancer subtypes. Boxplots of log2 FPKM values for 6 splicing factors in the discovery data set by subtype.
(TIF)

S9 Fig siRNA knockdown of splicing factors is confirmed by qrtPCR. Following transfection of siRNA, expression levels of splicing factors were quantified by qrt-PCR, normalized using TUBB, and compared to scrambled control siRNA.
(TIF)

S10 Fig Genes affected by knockdown of splicing factors show significant overlap with subtype-specific isoforms. (TIF)

S11 Fig Genes affected by knockdown of splicing factors show significant overlap with ER binding sites. (TIF)

S12 Fig Genes affected by knockdown of splicing factors show significant overlap with PR binding sites. (TIF)

S1 Table Sample Demographics University of Chicago Cohort. (XLS)

S2 Table Summary of Mapped Reads Statistics. (XLS)

S3 Table Differential Gene and Isoform Expression Data, Discovery UC Cohort. (TXT)

S4 Table Summary of Gene Set Enrichment Analysis for UC Discovery Cohort. (XLS)

S5 Table List of Splicing Factor Genes. (TXT)

S6 Table Subtype-specific Splice Factor expression. (XLS)

## References

[1] Jemal A, Siegel R, Xu J, Ward E. Cancer Statistics, 2010. CA Cancer J Clin. 2010; caac.20073. 10.3322/caac.20073 20610543

[2] Nguyen PL, Taghian AG, Katz MS, Niemierko A, Abi Raad RF, Boon WL, et al Breast cancer subtype approximated by estrogen receptor, progesterone receptor, and HER-2 is associated with local and distant recurrence after breast-conserving therapy. J Clin Oncol Off J Am Soc Clin Oncol. 2008;26: 2373–2378. 10.1200/JCO.2007.14.4287 18413639

[3] Piccart-Gebhart MJ, Procter M, Leyland-Jones B, Goldhirsch A, Untch M, Smith I, et al Trastuzumab after adjuvant chemotherapy in HER2-positive breast cancer. N Engl J Med. 2005;353: 1659–1672. 10.1056/NEJMoa052306 16236737

[4] Romond EH, Perez EA, Bryant J, Suman VJ, Geyer CE, Davidson NE, et al Trastuzumab plus adjuvant chemotherapy for operable HER2-positive breast cancer. N Engl J Med. 2005;353: 1673–1684. 10.1056/NEJMoa052122 16236738

[5] Effects of chemotherapy and hormonal therapy for early breast cancer on recurrence and 15-year survival: an overview of the randomised trials. Lancet. 2005;365: 1687–1717. 10.1016/S0140-6736(05)66544-0 15894097

[6] Freedman GM, Anderson PR, Li T, Nicolaou N. Locoregional recurrence of triple-negative breast cancer after breast-conserving surgery and radiation. Cancer. 2009;115: 946–951. 10.1002/cncr.24094 19156929 PMC2993502

[7] Kaplan HG, Malmgren JA. Impact of triple negative phenotype on breast cancer prognosis. Breast J. 2008;14: 456–463. 10.1111/j.1524-4741.2008.00622.x 18657139

[8] Sorlie T, Tibshirani R, Parker J, Hastie T, Marron JS, Nobel A, et al Repeated observation of breast tumor subtypes in independent gene expression data sets. Proc Natl Acad Sci U S A. 2003;100: 8418–8423. 10.1073/pnas.0932692100 12829800 PMC166244

[9] Goehe RW, Shultz JC, Murudkar C, Usanovic S, Lamour NF, Massey DH, et al hnRNP L regulates the tumorigenic capacity of lung cancer xenografts in mice via caspase-9 pre-mRNA processing. J Clin Invest. 2010;120: 3923–3939. 10.1172/JCI43552 20972334 PMC2964989

[10] Moore MJ, Wang Q, Kennedy CJ, Silver PA. An Alternative Splicing Network Links Cell-Cycle Control to Apoptosis. Cell. 2010;142: 625–636. 10.1016/j.cell.2010.07.019 20705336 PMC2924962

[11] Sveen A, Kilpinen S, Ruusulehto A, Lothe RA, Skotheim RI. Aberrant RNA splicing in cancer; expression changes and driver mutations of splicing factor genes. Oncogene. 2016;35: 2413–2427. 10.1038/onc.2015.318 26300000

[12] Adler AS, McCleland ML, Yee S, Yaylaoglu M, Hussain S, Cosino E, et al An integrative analysis of colon cancer identifies an essential function for PRPF6 in tumor growth. Genes Dev. 2014;28: 1068–1084. 10.1101/gad.237206.113 24788092 PMC4035536

[13] Okumura N, Yoshida H, Kitagishi Y, Nishimura Y, Matsuda S. Alternative splicings on p53, BRCA1 and PTEN genes involved in breast cancer. Biochem Biophys Res Commun. 2011;413: 395–399. 10.1016/j.bbrc.2011.08.098 21893034

[14] Brown RL, Reinke LM, Damerow MS, Perez D, Chodosh LA, Yang J, et al CD44 splice isoform switching in human and mouse epithelium is essential for epithelial-mesenchymal transition and breast cancer progression. J Clin Invest. 2011;121: 1064–1074. 10.1172/JCI44540 21393860 PMC3049398

[15] Shapiro IM, Cheng AW, Flytzanis NC, Balsamo M, Condeelis JS, Oktay MH, et al An EMT–Driven Alternative Splicing Program Occurs in Human Breast Cancer and Modulates Cellular Phenotype. PLoS Genet. 2011;7: e1002218 10.1371/journal.pgen.1002218 21876675 PMC3158048

[16] Yao R, Jiang H, Ma Y, Wang L, Wang L, Du J, et al PRMT7 Induces Epithelial-to-Mesenchymal Transition and Promotes Metastasis in Breast Cancer. Cancer Res. 2014;74: 5656–5667. 10.1158/0008-5472.CAN-14-0800 25136067

[17] Mortazavi A, Williams BA, McCue K, Schaeffer L, Wold B. Mapping and quantifying mammalian transcriptomes by RNA-Seq. Nat Methods. 2008;5: 621–628. 10.1038/nmeth.1226 18516045 PMC13303166

[18] Marioni JC, Mason CE, Mane SM, Stephens M, Gilad Y. RNA-seq: an assessment of technical reproducibility and comparison with gene expression arrays. Genome Res. 2008;18: 1509–1517. 10.1101/gr.079558.108 18550803 PMC2527709

[19] Trapnell C, Pachter L, Salzberg SL. TopHat: discovering splice junctions with RNA-Seq. Bioinforma Oxf Engl. 2009;25: 1105–1111. 10.1093/bioinformatics/btp120 PMC2672628 19289445

[20] Trapnell C, Williams BA, Pertea G, Mortazavi A, Kwan G, van Baren MJ, et al Transcript assembly and quantification by RNA-Seq reveals unannotated transcripts and isoform switching during cell differentiation. Nat Biotechnol. 2010;28: 511–515. 10.1038/nbt.1621 20436464 PMC3146043

[21] Parker JS, Mullins M, Cheang MCU, Leung S, Voduc D, Vickery T, et al Supervised risk predictor of breast cancer based on intrinsic subtypes. J Clin Oncol Off J Am Soc Clin Oncol. 2009;27: 1160–1167. 10.1200/JCO.2008.18.1370 PMC2667820 19204204

[22] Subramanian A. From the Cover: Gene set enrichment analysis: A knowledge-based approach for interpreting genome-wide expression profiles. Proc Natl Acad Sci. 2005;102: 15545–15550. 10.1073/pnas.0506580102 16199517 PMC1239896

[23] Dennis G, Sherman BT, Hosack DA, Yang J, Gao W, Lane HC, et al DAVID: Database for Annotation, Visualization, and Integrated Discovery. Genome Biol. 2003;4: R60–R60. PMC193660 12734009

[24] Weinberg F, Hamanaka R, Wheaton WW, Weinberg S, Joseph J, Lopez M, et al Mitochondrial metabolism and ROS generation are essential for Kras-mediated tumorigenicity. Proc Natl Acad Sci. 2010;107: 8788–8793. 10.1073/pnas.1003428107 20421486 PMC2889315

[25] David CJ, Manley JL. Alternative pre-mRNA splicing regulation in cancer: pathways and programs unhinged. Genes Dev. 2010;24: 2343–2364. 10.1101/gad.1973010 21041405 PMC2964746

[26] Gold B, Kirchhoff T, Stefanov S, Lautenberger J, Viale A, Garber J, et al Genome-wide association study provides evidence for a breast cancer risk locus at 6q22.33. Proc Natl Acad Sci U S A. 2008;105: 4340–4345. 10.1073/pnas.0800441105 18326623 PMC2393811

[27] Sablina AA, Chen W, Arroyo JD, Corral L, Hector M, Bulmer SE, et al The tumor suppressor PP2A Abeta regulates the RalA GTPase. Cell. 2007;129: 969–982. 10.1016/j.cell.2007.03.047 17540176 PMC1945132

[28] Fushimi K, Ray P, Kar A, Wang L, Sutherland LC, Wu JY. Up-regulation of the proapoptotic caspase 2 splicing isoform by a candidate tumor suppressor, RBM5. Proc Natl Acad Sci. 2008;105: 15708–15713. 10.1073/pnas.0805569105 18840686 PMC2572934

[29] Logette E, Wotawa A, Solier S, Desoche L, Solary E, Corcos L. The human caspase-2 gene: alternative promoters, pre-mRNA splicing and AUG usage direct isoform-specific expression. Oncogene. 2003;22: 935–946. 10.1038/sj.onc.1206172 12584573

[30] Koboldt DC, Fulton RS, McLellan MD, Schmidt H, Kalicki-Veizer J, McMichael JF, et al Comprehensive molecular portraits of human breast tumours. Nature. 2012;490: 61–70. 10.1038/nature11412 23000897 PMC3465532

[31] Hua S, Kittler R, White KP. Genomic antagonism between retinoic acid and estrogen signaling in breast cancer. Cell. 2009;137: 1259–1271. 10.1016/j.cell.2009.04.043 19563758 PMC3374131

[32] Eswaran J, Horvath A, Godbole S, Reddy SD, Mudvari P, Ohshiro K, et al RNA sequencing of cancer reveals novel splicing alterations. Sci Rep. 2013;3. 10.1038/srep01689 PMC3631769 23604310

