# Supplementary figures and images for: Robust stratification of breast cancer subtypes using differential patterns of transcript isoform expression
Source: PLoS Genet. 2017 Mar 6;13(3):e1006589. doi: 10.1371/journal.pgen.1006589 (PMC5367891; doi:10.1371/journal.pgen.1006589)

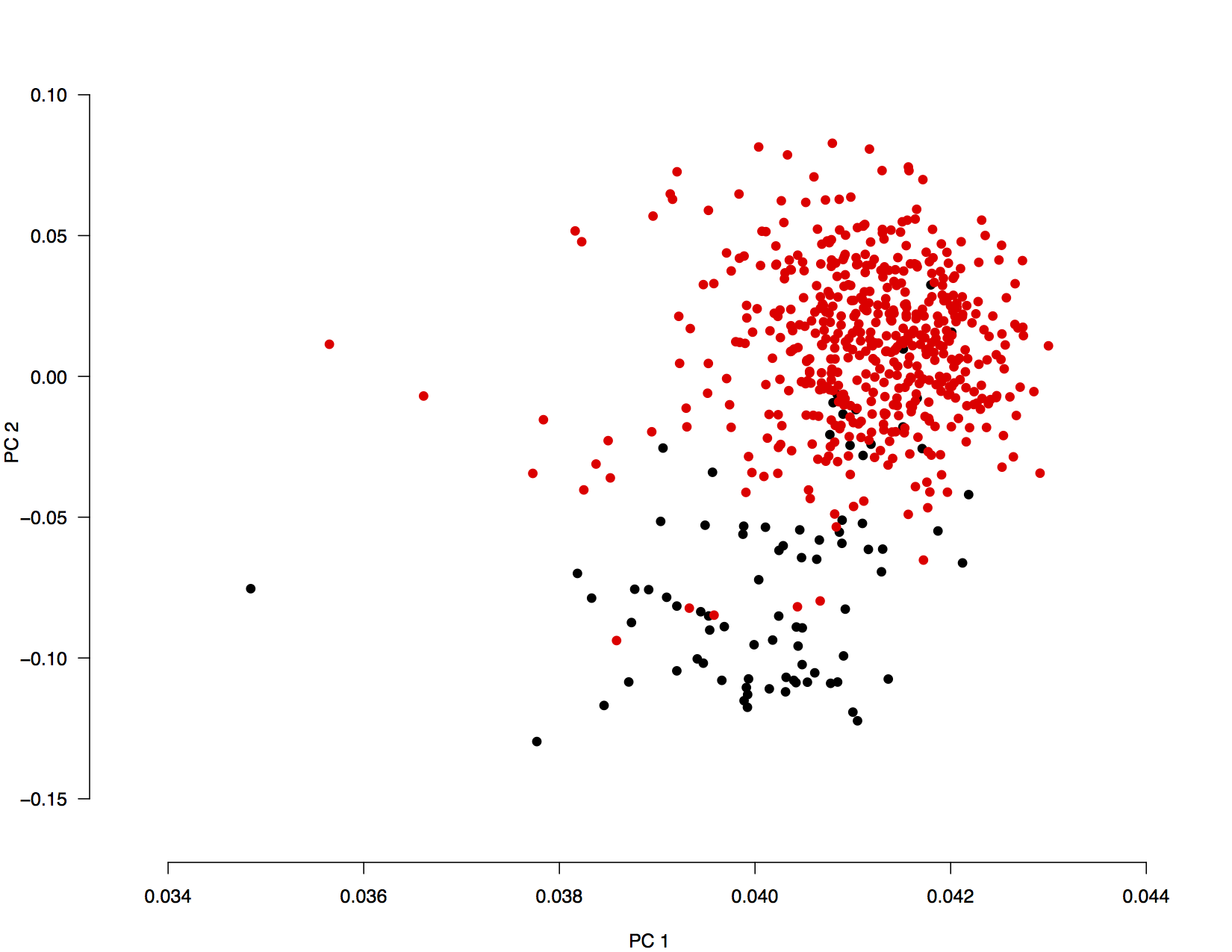

Supplement: S4 Fig — First two principal components derived from RefSeq gene RNAseq FPKM expression levels for single isoform genes only. TCGA samples are segregated by breast cancer subtype. (TIF) [file pgen.1006589.s004.tif]

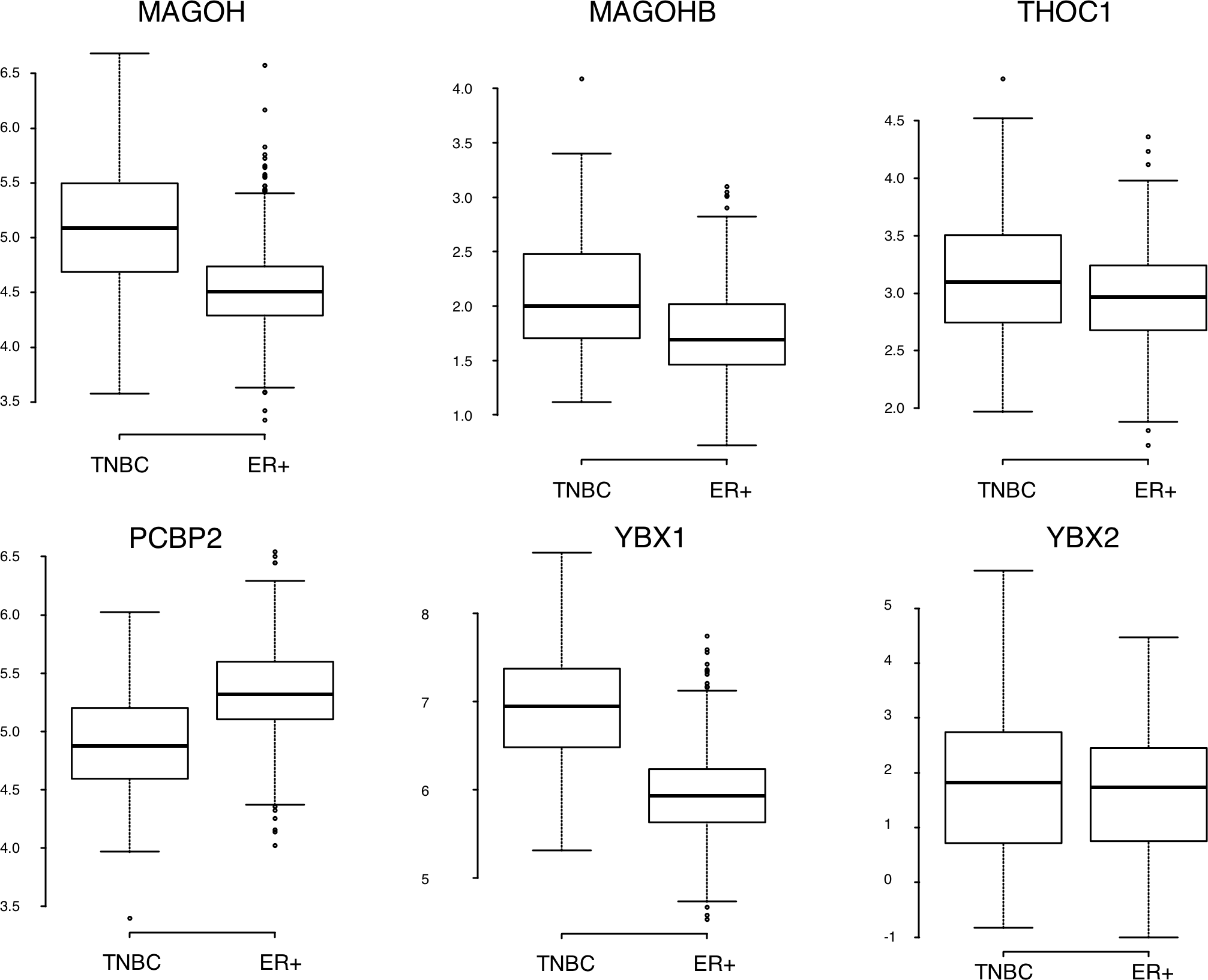

Supplement: S8 Fig — Boxplots of log2 FPKM values for 6 splicing factors in the discovery data set by subtype. (TIF) [file pgen.1006589.s008.tif]
